# On the Logical Design of a Prototypical Data Lake System for Biological Resources

**DOI:** 10.3389/fbioe.2020.553904

**Published:** 2020-09-29

**Authors:** Haoyang Che, Yucong Duan

**Affiliations:** College of Information Science and Technology, Hainan University, Haikou, China

**Keywords:** data lake, DIKW, biological resources, unstructured data, XAI, explainability, interpretability

## Abstract

Biological resources are multifarious encompassing organisms, genetic materials, populations, or any other biotic components of ecosystems, and fine-grained data management and processing of these diverse types of resources proposes a tremendous challenge for both researchers and practitioners. Before the conceptualization of data lakes, former big data management platforms in the research fields of computational biology and biomedicine could not deal with many practical data management tasks very well. As an effective complement to those previous systems, data lakes were devised to store voluminous, varied, and diversely structured or unstructured data in their native formats, for the sake of various analyses like reporting, modeling, data exploration, knowledge discovery, data visualization, advanced analysis, and machine learning. Due to their intrinsic traits, data lakes are thought to be ideal technologies for processing of hybrid biological resources in the format of text, image, audio, video, and structured tabular data. This paper proposes a method for constructing a practical data lake system for processing multimodal biological data using a prototype system named ProtoDLS, especially from the explainability point of view, which is indispensable to the rigor, transparency, persuasiveness, and trustworthiness of the applications in the field. ProtoDLS adopts a horizontal pipeline to ensure the intra-component explainability factors from data acquisition to data presentation, and a vertical pipeline to ensure the inner-component explainability factors including mathematics, algorithm, execution time, memory consumption, network latency, security, and sampling size. The dual mechanism can ensure the explainability guarantees on the entirety of the data lake system. ProtoDLS proves that a single point of explainability cannot thoroughly expound the cause and effect of the matter from an overall perspective, and adopting a systematic, dynamic, and multisided way of thinking and a system-oriented analysis method is critical when designing a data processing system for biological resources.

## Introduction

Biological resources encompass a vast range of organisms (and parts thereof), the genetic materials they contain (known more specifically as genetic resources), and any other biological components of a population or ecosystem that has an actual or potential use or value for human beings. The digitization of biological resources has created a large volume of biological big data; however, those data are possibly coming from multiple sources and are heterogeneous, and in order to make an actionable decision, there needs to be a trustful data integration and an integrated analytical solution. Research scientists in bioinformatics around the world have pulled all out of their efforts to collaboratively solve the challenging problems (Afgan et al., 2016; da Veiga Leprevost et al., 2017; Bussery et al., 2018). A data lake is claimed to be capable of fulfilling descriptive analytics, exploratory analytics, and confirmatory factor analytics requirements in other application fields, yet it has not been introduced in bioinformatics or genetics to store large quantities of biological resource data or experimental data in a massive way.

As a newly emerging paradigm of modern data architectures, the data lake radically simplifies the enterprise-wide data infrastructure, and it is expected to accelerate technological innovation alongside with the deep penetration of artificial intelligence and machine learning capabilities into every industrial and social sector. In the past, almost all of the data involved in the operational products and the decision-making products come from structured data stored in the back-end databases or data warehouses, or semi- or unstructured data crawled from the Web, and nowadays, many innovative products are embedding AI in the unstructured data format of computer vision, speech recognition, and text mining. These new requirements differ a great deal from the requirements emerging in the era of data warehouses, which need a structured, subject-oriented, and relational database claiming to hold a single view of data without data silos (Stein and Morrison, 2014). For example, over the years, Irvine Medical Center, University of California, had accumulated a pile of patient health records owned by one million inpatients and outpatients. These different types of data included online spreadsheet data, semi-structured medical reports, unstructured prescriptions, and radiology images from its radio department. The medical center had to store, integrate, and access the big data, so they chose to use a Hadoop distribution as their initial data lake infrastructure, for the benefit of Hadoop open-source software stacks and low-price commodity hardware clusters (Stein and Morrison, 2014). When it comes to the processing of biological resources, as research institutions, labs, and pharmaceutical plants increasingly use mobile apps and cloud services, the application scenarios will be somewhat similar to what they have experienced at the UC Irvine Medical Center.

Due to their intrinsic traits, data lakes are thought to be ideal technologies for processing of hybrid biological resources in the format of text, image, audio, video, and structured tabular data. Unfortunately, facing with these voluminous and heterogeneous data, current data lake proposals cannot afford the system complexity and high tolerance for human errors, due mostly to their incipient design and low explainability. However, some research directions and application scenarios have received special attention on the explainability due to their specialties and critical states, especially those in medicine and pharmacy pertaining to human lives where decisions are literally a matter of life or death. Biology as a discipline also concerns much on the explainability of biological phenomena and effects. Thus, the data management of biological resources urgently needs to solve the following two problems: (i) efficient and effective management of heterogeneous data from multiple sources and (ii) reasonable explanation of applications running on the platform in terms of the overall system design. Usually, explainability cares more about the Explainable Artificial Intelligence and Machine Learning (XAI and ML) algorithm (Samek et al., 2017) and recommender systems (Schafer et al., 1999; Zhang et al., 2014). However, we consider that explainability is a very broad term that still includes engineering-related aspects like the data/information/knowledge/wisdom spectrum, or DIKW (Duan et al., 2019), network architecture, and development language, human-related aspects like human faults, and cognitive psychology, not just algorithm and mathematics-related aspects. A single point of explainability cannot thoroughly expound the cause and effect of the matter from an overall perspective; we must adopt a systematic view and system-oriented analysis method.

Also, the data lake approaches may learn lessons and experiences from other similar approaches, which are possibly coming from different application domains, for example, the virtual research environment approaches (Assante et al., 2019; Houze-Cerfon et al., 2019; Remy et al., 2019; Albani et al., 2020). As is known to all, the integration of domain knowledge from different application domains will bring different perspectives to the data lake solutions.

Consequently, we propose in this paper the following:

1. to construct a practical data lake system for processing multimodal biological data using a prototype system named ProtoDLS;
2. to adopt a horizontal pipeline to ensure the intra-component explainability factors from data acquisition to data presentation, and a vertical pipeline to ensure the inner-component explainability factors including mathematics, algorithm, execution time, memory consumption, network latency, and sampling size.

In order to better understand the meaning of explainability from the outset, in here we give a brief definition of explainability and interpretability (Adadi and Berrada, 2018; Arrieta et al., 2020).

**Definition 1 Explainability** denotes an account of the system, its workings, and the implicit and explicit knowledge it uses to arrive at conclusions in general and the specific decision at hand, which is sensitive to the end-user’s understanding, context, and current needs.

**Definition 2 Interpretability** denotes the extent to which a cause and effect can be observed within a system. Or, to put it in another way, it is the extent to which you are able to predict what is going to happen, given a change in input or algorithmic parameters.

In the context of this article, explainability and interpretability are used interchangeably.

The reminder of this paper is organized as follows. Section “Related Work” briefly surveys the current *status quo* of data lakes and XAI, as well as the research development of data management approaches in the field of bioinformatics, genetics, and phenomics. Section “The Prototype Architecture” presents the overall architecture of our prototype system, i.e., ProtoDLS and describes the specific features of each key component. Sections “Horizontal Pipeline” and “Vertical Pipeline” elucidate the detailed design especially from the point view of explainability in a horizontal and vertical pipeline, respectively. Section “Project Progress and Discussion” discusses the current project progress and makes a contrast between ProtoDLS and emerging data lake systems. In closing, Section “Conclusion and Future Work” comes to a conclusion of the paper and outlines future development directions about ProtoDLS.

## Related Work

In 2010, James Dixon, CTO of Pentaho (2020), firstly proposed the concept of data lake in one blog post, as a way trying to store voluminous and diversely structured data in their native formats, in an evolutionary storage place allowing later detailed analyses (Dixon, 2010). Although the concept was first coined in early 2010, academia adopted it a couple of years later. Until now, there has been no well-accepted definition of what a data lake is, and the corresponding underlying features vary differently according to the real-world contexts. Some early research advancements on data lakes for a time were ever bound up with on-demand data models, or widely called schema-on-read models (also known as late binding models) (Fang, 2015; Miloslavskaya and Tolstoy, 2016). The key reason for adopting the schema-on-read model in data lakes lies in the bulk workloads of manual schema extraction, which is inoperable in the face of machine learning tasks, especially deep learning tasks. At the same time, Suriarachchi and Plale (2016) found that with the continuing growth of data in top gear, a data swamp will soon appear from a meant-to-be data lake without the guidance of a clear-cut schema. Thus, to ensure data accessibility, exploration, and exploitation, an efficient and effective metadata system becomes an indispensible component in data lakes (Quix et al., 2016). Yet, most of the research work on data lakes still concentrate on structured data, or semi-structured data only (Farid et al., 2016; Farrugia et al., 2016; Madera and Laurent, 2016; Quix et al., 2016; Klettke et al., 2017). So far, unstructured data have not received enough consideration in the relevant research literature, while more often than not unstructured heterogeneous data occur frequently (Miloslavskaya and Tolstoy, 2016). Multimodality in data lake systems is estimated to come under the spotlight in the next research wave.

Almost at the same time, with the development of big data and deep learning, especially since the totemic year of 2012, AI algorithms have attained or surpassed the limits of human beings in many areas like chess games and drug discovery, which were computationally unimaginable in early years (Lecun et al., 2015). However, some black-box models like random forest (Breiman, 2001), GBDT (Friedman, 2001), and deep learning (Lecun et al., 2015) have extraordinarily complex inner working mechanisms and inexplicable outer input–output mappings. Even for a senior graduate student, to fully understand the rationale of a black-box model will cost him several days and make him go through a painful process of a conscientious manual formula derivation and a time-consuming experimental verification. The problem with these models is that they are devoid of transparency and explainability, although they will nearly gain superior performance after careful fine-tuning. In the healthcare and medical field, that would become a big problem since applications in these demanding fields require a full-fledged explanation of model rationales. Thus, research efforts in these fields have witnessed a burst of articles and papers in explainable artificial intelligence (XAI) (Došilović et al., 2018). Since XAI methods have extensive application scenarios, a full survey of XAI research and development is a difficult task to accomplish. On a large scale, the related research topics in XAI can be roughly divided into two major categories: integrated approaches and *post-hoc* approaches.

The integrated approaches usually keep an eye on the transparency factors, and transparency is a required means for the protection of human rights from unfairness and discrimination (Edwards and Veale, 2017). Similar to the idea, transparent models are expected to be both explainable and interpretable. As one of its subbranches, pure transparent approaches restrict the model choices to the model families that are considered transparent. For example, Himabindu et al. (2016) ever proposed a method to use separate if-then rules to effectively interpret decision-making sets. Based on region-specific predictive models, Wang et al. (2015) proposed an oblique treed sparse additive model, which exchanges a modest measure of interpretability for accuracy, but in SVM and some other non-linear models, it gains a satisfying degree of accuracy. As another subbranch, hybrid approaches combine pure transparent models and black-box models to get a balance between interpretability and performance. To develop internal rating models for banks, Gestel et al. (2005) used a progressive method balancing the requirements of predictability and interpretability.

*Post-hoc* approaches will not impact the model performance since it extracts information from the already learned model. Usually, *post-hoc* approaches are used in cases where model mechanisms are too complex to explain. For example, as for explainable recommendation, two diverse models generate recommendations and explanations, respectively. After the genuine recommendations have been performed, an explanation model independent of the recommendation algorithms will provide explanations for the recommendation model carried out just a while ago (so it is called as “*post-hoc*”). Likewise, to provide a *post-hoc* explainability for recommendations, Peake and Wang also presented a data mining method with several association rules (Peake and Wang, 2018). In addition to recommendation, *post-hoc* approaches were also used in image recognition and text classification. To find out model defects in these fields, with the aid of several elastic nets, Guo et al. (2018) augmented a Bayesian regression mixture model and extracted explanations for a target model through global approximation.

XAI has received relatively little attention in the field of bioinformatics and biology, but ontology-based data management in this line has gleaned quite a few studies. In phenomics research, aiming to support adequate collaboration between teamworkers, Li Y.F. et al. (2010) presented PODD, a data reservoir based on ontology. Like its big brother, genomics, phenomics research uses imaging devices and measurement apparatuses to acquire vast amounts of generated data, which are subsequently used for analysis. Thus, in phenomics research, there are key challenges for data management of large amounts of raw data (image, video, raw text). Meanwhile, in genomics, Ashburner et al. (2000) constructed the famous Gene Ontology (Gene Ontology, 2020), a well-established and structured tool to represent gene ontology categories and terms, which has been successfully used for many years by researchers. The Gene Ontology includes three independent ontologies, molecular function ontology, cellular component ontology, and biological process, and can be used for all eukaryotes, even as we are gaining more knowledge of protein and gene functions in cells (Ashburner et al., 2000). The Bio2RDF (2020) project declares to transform silos of life science data into a globally distributed network of linked data for biomedical knowledge translation and discovery. Up until now, Bio2RDF has accumulated 378 datasets, including Bio2RDF:Drugbank and Bio2RDF:Pubmed. The EBI RDF platform (Ebi Rdf, 2020) claims to bring together a number of EMBL-EBI resources that provide access to their data using Semantic Web technologies. As a well-accepted exchange language, BioPAX (Biological Pathway Exchange) aims to enable integration, exchange, visualization, and analysis of biological pathway data (Demir et al., 2010). OntoLingua provides a distributed collaborative environment to browse, create, edit, modify, and use ontologies (OntoLingua Server, 2020). The BioSchemas project develops different types of schemas for the exchange of biological data and aims to reuse existing standards and reach consensus among a wide number of life science organizations (BioSchemas, 2020). Although closed, W3C’s HCLS (Health Care and Life Sciences) group has done a great deal of work to use Semantic Web technologies across health care, life sciences, clinical research, and translational medicine. Amid questions about its feasibility and availability, IBM Watson brings to customers a cognitive computing platform which can understand, reason, and learn from a magnitude of unstructured medical literature, patents, genomics, and chemical and pharmacological data (Ying et al., 2016).

Apart from ontology research in biology, at different times, a collection of databases such as scientific publications (PubMed, 2020), genes (Ensembl, 2020), proteins (UniProt, 2020), and gene expression data (Ebi ArrayExpress, 2020; Gene Expression Atlas, 2020; GEO, 2020) have ever been created in order to store big quantities of bio-data for the purpose of refining and for systematic scientific research work. These data storage platforms have seldom based on data lakes since data lakes are not as mature as commercial databases and data warehouses; neither are open-source data management solutions like Hadoop software stack.

At the intersection of data lakes and explainability, research on explainable data lakes still remains unexplored. Also, for now, barely little literature in the field of data lakes has discussed explainability systematically. This paper tries to fill the gap between data lakes and explainability from a systematic view, not just a XAI view, and to borrow knowledge and experience from the research development on XAI and data lakes.

## The Prototype Architecture

**FIGURE 1:**
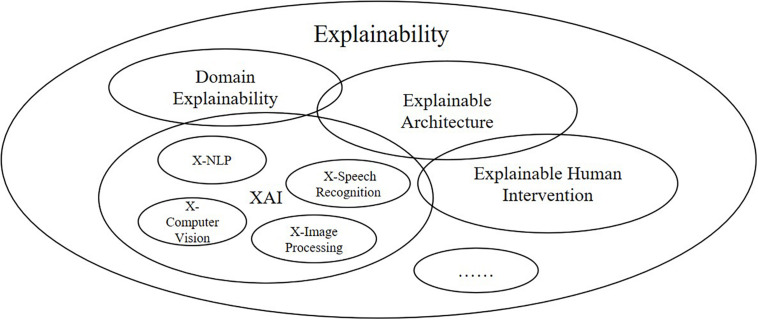
Explainability is a comprehensive concept and XAI is just an aspect of explainability.

In this section, we will present the overall architecture of ProtoDLS (Prototypical Data Lake System for Biological Resources) we have designed. In the field of bioinformatics and genetics, ProtoDLS intends to answer the explainability problem in a systematic, dynamic, and multisided view instead of an isolated, static, and one-sided view. In order to explain certain questions about data, metrics, rules, and business objectives, ProtoDLS insists that only every component and module is self-explanatory itself, the unhindered explainability can be thoroughly implemented in the system level as a whole, and only after that, explainability can take real effect and solve real-world problems. ProtoDLS also disbelieves a single point of explainability such as XAI for that XAI also has input into, output out of, and interactions with other components, modules, or even machine learning algorithms in a data lake system as in Figure 1.

**FIGURE 2:**
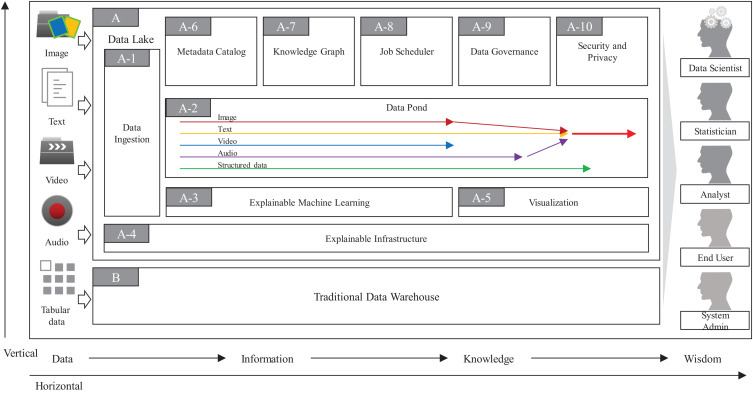
The overall architecture of ProtoDLS.

As in Figure 2, the overall architecture of ProtoDLS can be roughly divided into two major components: **Data Lake** (DL, A in Figure 2) and **Traditional Data Warehouse** (TDW, B in Figure 2). Initially debuted as a substitute for data marts in the topmost tier of data warehouses, data lakes have exhibited a relationship of complement to data warehouses rather than a competitive relationship with data warehouses. The complementary strengths and challenges between them in recent years also suggest the urgent needs to exchange ideas on opportunities, challenges, and cutting-edge techniques within them. In ProtoDLS, TDW is usually used to cleanse, integrate, store, and analyze the processed, trusted, and well-structured data or semi-structured data like website logs. Raw data is always discarded or stored in a NAS/SAN/Cloud storage area. TDW and DL transfers data back and forth; sometimes, DL can serve as a staging area for TDW, and vice versa. DL stores raw data in any format and outputs the deeply analyzed results in a schematic format to TDW for visualization, reporting, and *ad hoc* query. TDW also outputs some structured data to DL as its metadata and elementary elements. The detailed data flow between them is stored in **Metadata Catalog** (MC, A-6 in Figure 2) of DL for later explanation and traceability.

The **Data Ingestion** (DI, A-1 in Figure 2) component of ProtoDLS provides an appropriate data extraction, integration, transformation, and load mode for multiple heterogeneous data sources. DI has the following features:

1. **Data source configuration:** to support multiple data sources, including but not limited to TDW, databases, flat files, message queues, and protocol datagrams.
2. **Data collection:** to support the collection actions of the corresponding data source, and complete data structure analysis, data cleaning, data transforming, data normalization, data format standardization, etc.
3. **Data synchronization:** to support data synchronization to other data sources, including necessary cleaning, processing, and transforming.
4. **Data distribution:** to support data sharing and distribution, and publish data in various forms (object stores, APIs, etc.).
5. **Data preprocessing:** to support data encryption, desensitization, standardization, and other particular processing logic.

The **Explainable Infrastructure** (EI, A-4 in Figure 2) component of ProtoDLS is slightly different from the traditional infrastructure layer. EI is also composed of network unit, storage unit, and computing unit. The generated data are all collected in these units, such as memory consumption by second, network latency by second, storage capacity by hour/day. The warning, alert, or other important system admin event will be triggered and displayed in an intuitive way, like using NLG (Natural Language Generation).

The **Visualization** (VI, A-5 in Figure 2) component of ProtoDLS empowers other components with visualization capabilities. With visualization, horizontal components and vertical modules can enhance explainability of DIKW flow (see below), statistical algorithms, and deep learning algorithms.

In one respect, the design of a data lake platform is fundamentally metadata driven, especially in terms of explainability. The **MC** component of ProtoDLS is very critical to explainability, since it globally stores all of the metadata generated locally in every component, of which type includes technical metadata, business metadata, and operational metadata. MC stores every and each data change and schema change. MC can represent metadata in tabular forms or human understandable sentences supported by the **Explainable Machine Learning** (EML, A-3 in Figure 2) component. MC provides a system-wide single point of truth for all kinds of users in ProtoDLS.

The **Knowledge Graph** (KG, A-7 in Figure 2) component of ProtoDLS visualizes knowledge entities and the relationships between the entities in a graph model. With the help of KG, EML, or any other component in ProtoDLS can extract named entity, relationship, and attributes from it. Knowledge representation, knowledge fusion, entity disambiguation, and knowledge/ontology reasoning in KG can enhance explainability in other components. The question and answer feature is critical to explainability, and KG can provide accurate and concise natural language abilities to aid it.

The **Job Scheduler** (JS, A-8 in Figure 2) component of ProtoDLS schedules jobs for execution (start, stop, terminate, invocate, replay, or sleep) at a specific time/date, or triggers jobs upon receiving some certain event, or records the execution orders of jobs and running status within jobs in DC. JS orchestrates the running jobs in ProtoDLS in a sequential or concurrent order, and its scheduling trigger scheme includes time-based, interval-based, and event-based.

In contrast to TDW, the biological data maintained in DL are more scattered, disordered, and schema-less, so it is more necessary to govern the data usability, availability, integrity, security, and flows in DL through the work of **Data Governance** (DG, A-9 in Figure 2), otherwise DL will gradually become corrupted and finally transforms into a data swamp. To efficiently and effectively drive data intelligence, DG is crucial and it is also one of the biggest challenges during the construction of DL. The core task of DG lies in improving the multimodal data quality by the aid of metadata management, data standard conformance, data lifecycle management, data security and privacy management, and data stewardship. Without the aid of DG, low-quality data will greatly lower the precision and recall of machine learning algorithms and thus will further restrict the interpretability and explainability of ProtoDLS as a whole.

The **Security and Privacy** (SP, A-10 in Figure 2) component of ProtoDLS deals with security and privacy issues since ProtoDLS will be flooded by the influx of numerous raw and unprocessed data, which will be very dangerous without some appropriate supervision, audit, and access control methods. Privacy preserving data mining can protect personal privacy data from leakage and damage, improve explainability, and reduce bias.

The **Data Pond** (DP, A-2 in Figure 2) component of ProtoDLS subdivides and processes the data exported by DI according to the incoming data format. ProtoDLS needs to provide a variety of data analysis engines to meet the needs of data computing. It needs to meet batch, real-time, streaming, and other specific computing scenarios. In addition, it also needs to provide access to massive data to meet the demand of high concurrency and improve the efficiency of real-time analysis. Heterogeneous data enters into DP according to the dispatch of JS. Initially, text data enter into text DP, and image data enter into image DP, and so on. When multimodality analysis is set, different types of data may enter into a hybrid DP, for example, text data and image data may enter into text-image DP for later coordinated processing. The partition of DP over data formats ensures the explainability and traceability in DP.

The EML component of ProtoDLS is responsible for executing NLP, image classification, video classification, audio recognition, and conventional machine learning and deep learning algorithms in an explainable way. The methods for explainability may include example illustration, analogy, visualization, model-agnostic, local approximation, or even human intervention.

Potentially, ProtoDLS has a wide range of platform users including system administrators, data scientists, statisticians, analysts, and ordinary end users, who have different explainability demands for ProtoDLS. For discovery and ideation, data scientists will currently focus more on the explainability of the black-box deep learning algorithms. Statisticians will pull all out to explore data patterns and identify data rules through tests, summaries, and higher-order statistics under some hypothesis. Data analysts may cost their efforts to explain the business intelligence metrics in their everyday life. System administrators will pay attention to the normal operation of ProtoDLS. When the system is down or a performance degradation occurs, EI in ProtoDLS will give system administrators an easy-to-understand explanation and system administrators will rephrase the explanation in less technical terms to other users of ProtoDLS, in order to mitigate the user anxieties and confusion. Ordinary end users usually are not technical experts in the abovementioned areas, and all they want is an easy-to-understand explanation. According to the explanation, they will make decisions and enact policies and rules. However, the requirement creates the most difficult part of explainability in ProtoDLS, since the generated explanation by the platform must be presented in an intuitive way prone to human understanding, without many technical terms or nomenclatures. ProtoDLS accumulates all the explanations in every component and module and ranks them in an important or critical order, and ProtoDLS will synthesize them into a paragraph that human can easily understand and accept. The training procedures will absorb insights and suggestions from experts in bioinformatics, genetics, and phenomics, in algorithms, in computer architecture, or even in cognitive psychology.

ProtoDLS aims to help researchers and practitioners in bioinformatics, genetics, and phenomics finish the following tasks in an explainable way:

1. Multimodality data governance over datasets collected from biological resources, including data standard conformance, data security enforcement, metadata management, data quality improvement, data stewardship, and data lifecycle management, with an aim to reduce the difficulties of data analytics without data lakes.
2. Multimodality data exploration and exploitation using cutting-edge machine learning, deep learning, and artificial intelligence techniques, on the basis of ingesting, aggregating, cleaning, and managing datasets maintained in ProtoDLS.
3. To generate new data dimensions based on the analysis of previous usage histories.
4. To create a centralized multimodality data repository for data scientists and data analysts, etc., which is conducive to the realization of a data service optimized for data transmission.

## Horizontal Pipeline

In ProtoDLS, the horizontal pipeline mainly concerns about when, where, how, by who, and to what degree the large amounts of instantly collected raw data are being transformed into meaningful information, useful knowledge, even insightful wisdom for all kinds of end users. In the horizontal direction, ProtoDLS is divided into several components according to specific functional requirements. Thus, ProtoDLS adopts a horizontal pipeline to ensure the intra-component (or subsystem-level) explainability across the horizontal landscape, from data acquisition, to data storage, all the way up to data processing, and finally to data presentation.

### Data, Information, Knowledge, Wisdom

**FIGURE 3:**
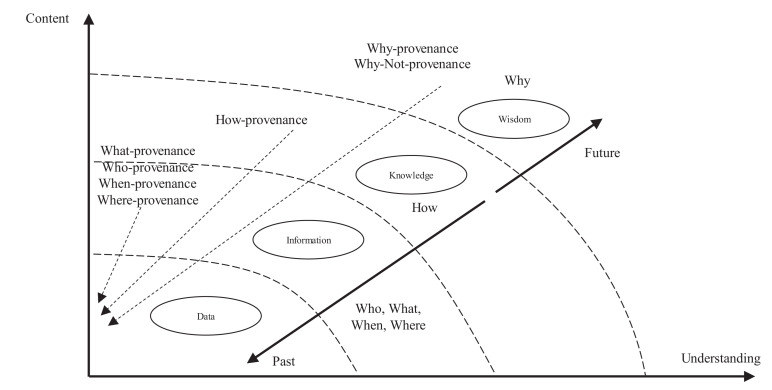
The DIKW provenance.

ProtoDLS observes and manages the data flow between horizontal units in light of the conceptual framework of DIKW (Duan et al., 2019). As seen in Figure 3, the DIKW model integrates data, information, knowledge, and wisdom in a set of related layers, each extending the ones underneath itself. The original observation and measurement activities obtain the raw data, in the format of image/text/video/audio, and the relationships between the raw data are analyzed accordingly to obtain the information, which in ProtoDLS is the integrated and formatted data from the raw data according to some ETL processing rules. The application of information in action produces knowledge, which in ProtoDLS is information applied to bioinformatics, genetics, and phenomics. Wisdom is concerned about the future, and it tries to understand things that have not been understood in the past, things that have not been done in the past.

Finding out how data, information, knowledge, and wisdom flow between components should act as the first step toward the complete explainability of a specific question posed toward ProtoDLS, since quick location of the need-to-explain points will be realized in terms of clear-and-cut DIKW flows.

ProtoDLS records any DIKW flow between every two components in MC in the following format: <Task_Name, Source_Component_Name | External_Source_Name, DIKW, Sink_Component_Name | External_Source_Name, Last Execution Time, Duration, Executed By >, i.e., DIKW flows from a source component or an external source into a sink component or flows out into an external source. MC can give back the DIKW flow path for any request from other components. System administrators can monitor and retrieve the DIKW flow in a single operational console. When incidents occur, DIKW flow monitoring capabilities can give admin teams a quick start and a good explanation for other platform users.

### DIKW Provenance

Provenance was a concept originated from the database community decades ago (Buneman et al., 2001). The emergence of data provenance, or data lineage, is that database or data warehouse users need to find out the data origin and the data evolution process, where they are coming from, where they are going, and what is happening to them; also, they need to frequently execute impact analyses in order to make sure that certain actions to be performed will affect the system in a controlled way and within a controlled range, or trace back data quality issues and errors till to their root causes as fast as they can. Or, on the other hand, many senior technical users like data scientists and data analysts tend to use datasets in isolation or in a team, which may quickly create some explicit or implicit upstream and downstream dependencies and chaining of dependent data processing. In this regard, the system or the platform need to cover a broad spectrum of workload scenarios like batch jobs, streaming jobs, mini-batch queries, *ad hoc* queries, deep learning training tasks, and support programming languages like R, Python, and Scala, and even new programming languages like Julia. To perform provenance on the data lake, we need DIKW provenance as an upgraded version in place of data provenance. With DIKW provenance, the ProtoDLS users can track and understand how DIKW flows across the platform at every stage, where DIKW resources are sourced from, and how they are being consumed, thus allowing users to develop trust and confidence in the platform, algorithms, infrastructure, and other inner working mechanisms of ProtoDLS.

Basically, DIKW provenance has the following categories:

1. What—provenance answers the question: what does this do?
2. Who—provenance answers the question: who did this?
3. When—provenance answers the question: when did this happen?
4. Where—provenance answers the question: where did this happen?
5. How—provenance answers the question: how the knowledge is worked out?
6. Why—provenance answers the question: why the result is working?
7. Why Not—provenance answers the question: why the result is not achieved?

**FIGURE 4:**
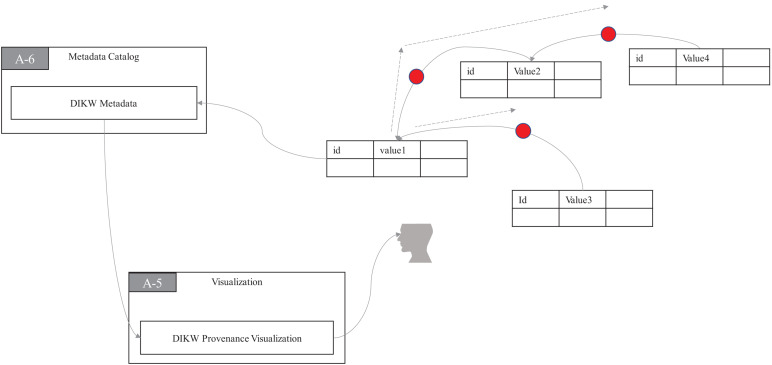
The DIKW provenance visualization.

With DIKW provenance, many questions related to the horizontal pipeline can be answered and thus explainability on a horizontal level will be achieved to some extent in ProtoDLS. As seen in Figure 4, the DIKW provenance flows can be recorded in a module named **DIKW Metadata** (DM) in MC, and the **DIKW Provenance Visualization** (DPV) module in VI is responsible for replaying the provenance flows in a reverse direction using animation.

## Vertical Pipeline

In ProtoDLS, the vertical pipeline concerns more about the explainability in every component rather than the intra-component explainability. For example, the fusion algorithm implemented in DP may require an explanation mainly in the scope of DP, rather than an explanation of the DIKW flows between DI and DP which has been explained and can be queried in the horizontal pipeline.

### Mathematics

For certain users, mathematics can be annoyingly inevitable when they conduct data analytics; however, mathematics is central to the area of computational biology and biomedical research. Some mathematical equations are relatively straightforward and easy to explain to the ordinary users, just like the famous SVD (Singular Value Decomposition) theorem:

$$M=U\,\Sigma \,V^{T}$$

Although the formula is simple in its form, the meaning behind it is quite wide and deep. That is, a seemingly simple formula also needs thorough explanation for ordinary end users. Furthermore, when stepping into the territory of machine learning, we will find that this area is glutted with so many cranky mathematical equations and formulas in all sorts of complex and profound algorithms, for example, convex optimization algorithms (Boyd et al., 2006) and the ELM (Extreme Learning Machine) algorithm (Huang et al., 2006), just like the following one excerpted from ELM:

$$\min\,L_{\mathrm{RELM}}=\frac{1}{2}\,{||\beta ||}^{2}+\frac{C}{2}\,{||Y-H\,\beta ||}^{2}$$

Some mathematical formulas are very hard to comprehend even by seasoned machine learning experts. Therefore, it is necessary to explain different mathematical formulas, even those seemingly ones in the system. To understand mathematical formulas is fundamental to understanding how a complex algorithm works as a whole. ProtoDLS thinks about the problem in three aspects:

1. to explain the interpretation of each symbol in the mathematical formula.
2. to explain the denotation of the entire mathematical formula including its user, context, etc.
3. to explain the connotation lying behind the entire mathematical formula.

**FIGURE 5:**
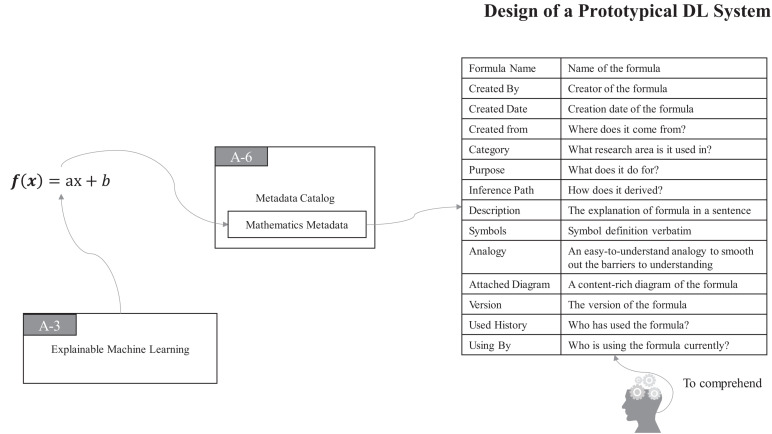
The MC component records all about mathematical formulas and formula inference processes.

Thus, in ProtoDLS, MC also records all of the bits and pieces about every mathematical formula and the associated formula derivation process in a particular region of it, namely, the **Mathematics Metadata** (MM) region, as seen in Figure 5.

With MM in MC, all the components and users of ProtoDLS can easily query, retrieve, and check in mathematics-related problems. At the same time, with the aid of VI, ProtoDLS can offer its users with an intuitive visualization presentation for better explainability.

### Algorithms

Likewise, algorithms, especially black-box deep learning algorithms are also hard to comprehend. There roughly exist two different cases when we are dealing with algorithms in practical applications:

1. Algorithms cannot be thoroughly understood by ordinary end users.
2. Algorithms cannot be clearly explained by current technical advancements, like black-box series of deep learning algorithms.

The first case can be somewhat eased by technical measures, like visualization and narrative storytelling. When it comes to the second case, explainability will soon become the bottlenecks of the whole platform. The methods we could adopt are reproducing algorithms and methods in the latest literature in ProtoDLS.

**FIGURE 6:**
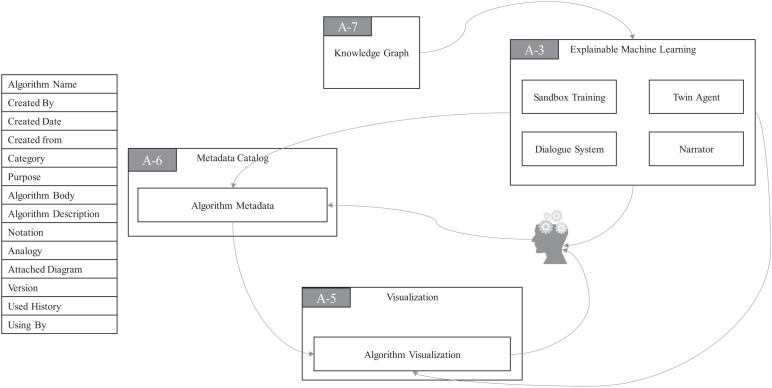
Algorithmic design for explainability in ProtoDLS.

**FIGURE 7:**
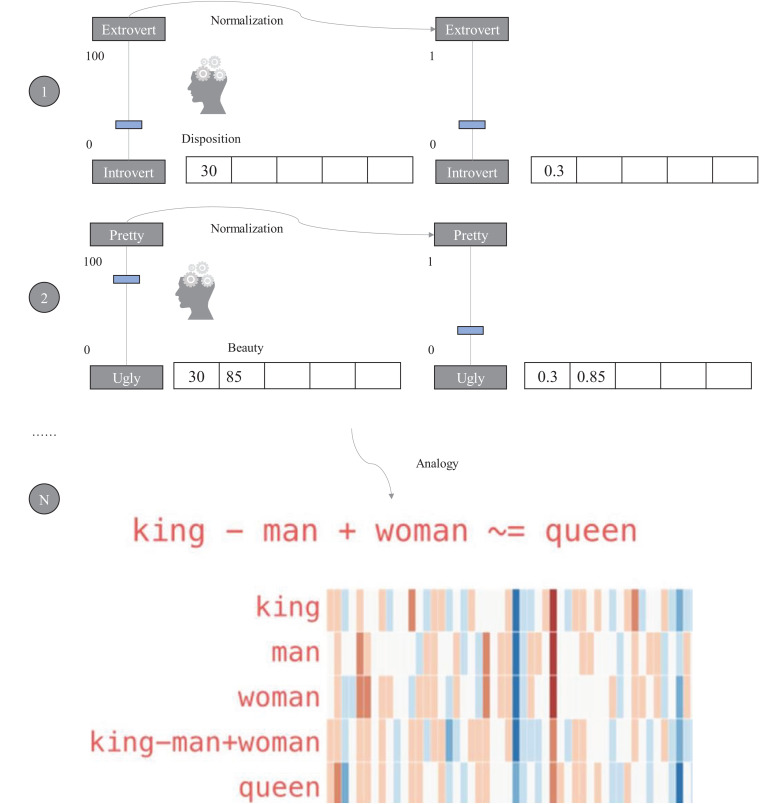
A narrative of the concept of word embedding.

As you can see in Figure 6, there is a module named **Algorithm Metadata** (AM) in MC. AM preserves all the information related to algorithms. The **Algorithm Visualization** (AV) module in VI plays a very special role in the algorithmic framework of ProtoDLS, since visualization is very critical to the explainability of algorithms, especially the deep learning algorithms. In the algorithmic framework, four modules exist in EML, i.e., **Sandbox Training** (ST), **Dialogue System** (DS), **Narrator** (NA), and **Twin Agent** (TA). At the same time, EML opens up a sandbox region in DP, specifically for model training, which has the following benefits: (i) algorithms can be trained and tested with real data sets in the production environment, and training the algorithm with the same data distribution will enhance the explainability; (ii) deployment from the sandbox region to the production environment is relatively straightforward; and (iii) the sandbox is isolated from the production environment and thus faults or halts of sandbox will not affect the production environment. The events that occurred in ST will go into the AM and be stored for later explanation. The dialogue bots behind DS interact with algorithm users with multiround natural language dialogues, with the help of KG. NA tells users how the algorithms work in a narrative storytelling mode. For example, it is well-known that beginners to NLP usually find it very hard to understand the concept of embedding. Embedding is a technique of mapping an object onto a vector. Without any explanation, this definition cannot be thoroughly understood by beginners. However, we may use a narrative of the algorithm to exchange for understandability, as seen in Figure 7. The idea of TA in EML is imitated by a reinforcement framework proposed by Wang et al. (2018). Sometimes, the machine learning algorithm is too complex to be thoroughly grasped. In such cases, we usually choose an explanation model to give *post-hoc* explanations about the real algorithm model. Based on this basic idea, the twin agents go further and select the best explanations about the algorithm model according to some reinforcement learning and adversarial learning rules. Drawing inspiration from this idea, TA simulates a reinforcement learning framework to enhance the explainability of some hard black-box algorithms.

### Engineering Explainability

In ProtoDLS, engineering factors of explainability consist of two major categories: the infrastructure (storage, memory, network, and computation) explainability and the software development explainability (programming language, process, thread, software methodology). The infrastructure explainability concentrates on the explanation of the running status of the infrastructure, like for example the question: how much memory do the underlying cluster nodes consume now? Meanwhile, the software development explainability concentrates on the explanation of program-related problems and questions.

EI uses a simple audit log to record all file access requests of the file system, intended to be easily written and non-intrusive. The log details include operation status (success, halted, failed, etc.), user name, client address, operation command, and operation directory. Through the audit log, system admins can view all kinds of operation status of EI in real time, track all kinds of warnings, errors, alerts, and incorrect operations, and execute some metric monitoring.

**FIGURE 8:**
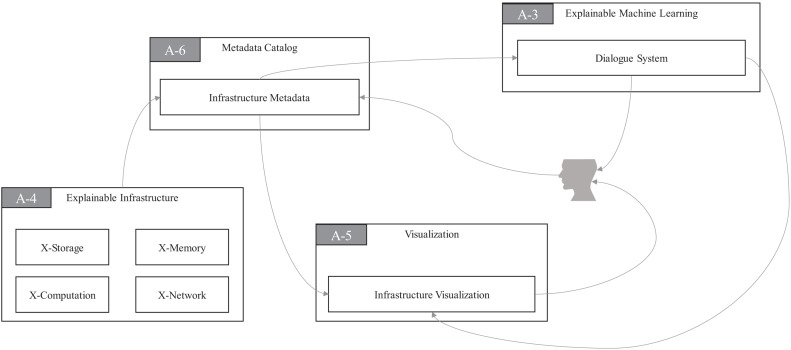
Infrastructure design for explainability in ProtoDLS.

At the same time, EI daemons will generate a series of monitoring logs. The monitoring log monitors and collects the measurable information of EI according to some predefined rules. For example, the following metrics will be collected by EI: the number of bytes written, the number of file blocks copied, and the number of requests from the client. EI daemons also monitor the network latency, memory consumption, and storage consumption. The X-Storage, X-Memory, X-Computation, and X-Network modules of EI continuously monitor their metrics and output the monitored metrics to the **Infrastructure Metadata** (IM) module of MC, respectively, as their names indicate. IM in MC transfers metrics to the **Infrastructure Visualization** (IV) module of VI to monitor the running status of EI on a visual interface for system administrators in real time on one side. On the other side, the DS module in EMI can asynchronously request metrics from IM to finish multiround natural language dialogue with users, as seen in Figure 8.

**FIGURE 9:**
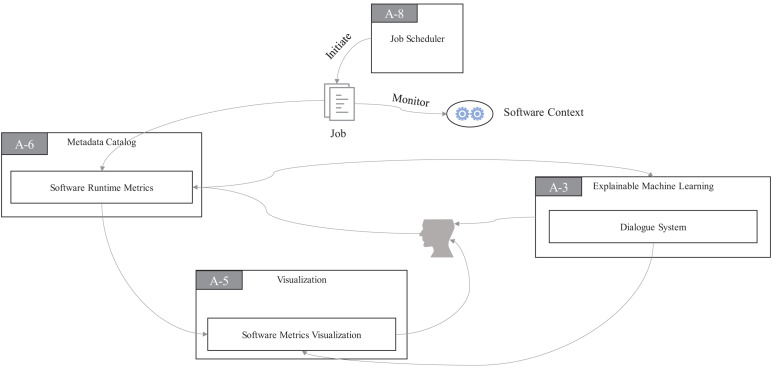
Software runtime status design for explainability in ProtoDLS.

Programming languages used for the computations also would affect our correct understanding of runtime contexts, algorithms, and running results. The runtime context of a process is composed of its program code, data structure, and hardware environment needed for program running. To collect the runtime context of programs, the JS component in ProtoDLS will start a job to monitor in real time, and it will store the monitored metrics in MC, as seen in Figure 9. Similarly, the collected metrics will store in a module named **Software Runtime Metrics** (SRM) in MC. SRM transfers the processed metrics to the **Software Metrics Visualization** (SMV) module of VI to monitor the running status of software contexts on a visual interface for software users or developers in real time on one side. On the other side, the DS module in EMI can request metrics from SRM to finish multiround natural language dialogue with software users or developers, as seen in Figure 9.

### Modality Explainability

ProtoDLS is created to support multimodality in the first place since it was chartered and initially designed for multimodal bioinformation processing for research purposes. Multimodality itself is a hard problem both in techniques and in applications, which causes several aspects of explainability needs. The explainability of multimodality lies in multimodal fusion. Multimodal fusion refers to the synthesis of information from two or more modalities for preprocessing. Multimodal fusion can be roughly divided into three types of fusion: early fusion, late fusion, and intermediate fusion.

The early fusion or data-level fusion combines multiple independent data sets into a single feature vector and then inputs it into a machine learning classifier. Because the early fusion of multimodal data often cannot fully utilize the complementarity of multimodal data, the original data of early fusion usually contain a great deal of redundant information. Therefore, early fusion methods are often combined with feature extraction methods to eliminate redundant information, such as principal component analysis (PCA) (Jolliffe, 2005), mRMR (Peng et al., 2005), and autoencoders (Vincent et al., 2008). In this regard, feature-level explainability largely determines the overall explainability of the fusion model.

The late fusion or decision-level fusion fuses the output scores of classifiers for decision-making trained by different modal data. The advantage of this method lies in that the errors of the fusion model come from different classifiers, while the errors from different classifiers are often separate and independent, which will not cause further accumulation of errors. Common late fusion methods include max fusion, average fusion, Bayes-based fusion, and ensemble learning fusion. As a typical representative of late fusion, ensemble learning is widely used in communication, computer recognition, speech recognition, and many other research fields. As a classical model-agnostic method, LIME (Local Interpretable Model-agnostic Explanations) will help to explain the late fusion of multimodality data (Ribeiro et al., 2018).

Intermediate fusion refers to the transformation of different modal data into high-dimensional feature expression, and then fusion in the middle layer of the model. Taking the neural networks as an example, the intermediate fusion first uses the neural network to transform the original data into high-dimensional feature representation and then obtains the commonalities of different modal data in high-dimensional space. One of the advantages of the intermediate fusion method is that it can flexibly choose the location where fusion happens.

The location where multimodal data fusion happens directly relates to the explainability. The late fusion method is a little bit easier to explain than the intermediate fusion and the early fusion. Moreover, the explainability also relates to the classifiers and is constrained by the explainability of the participated classifiers, which engenders new difficulties. In ProtoDLS, we treat modality explainability in a quite straightforward way, so we view the modality explainability independently and explain the participated algorithms or classifiers irrelevantly at first. In terms of its complexities and the current technical limitations, we leave the modality explainability as an open problem to be tackled in the future.

## Project Progress and Discussion

ProtoDLS started from an initial intent to build a platform supporting multimodal bioinformation processing for research and experiment purposes, with explainability in the heart of its design goals. In addition, some other minor design goals might include performance, robustness, security, privacy-preserving, and extensibility. However, meeting these design goals at the same time will greatly increase the complexity of system construction. Thus, we focus completely on the explainability of ProtoDLS in the first stage.

**FIGURE 10:**
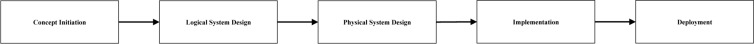
Software development process of ProtoDLS.

As seen in Figure 10, the whole software development process roughly includes the following phases: concept initiation, logical design, physical design, implementation, and deployment. Currently, we have just finished the logical system design and entered into the physical system design phase. During the phase, we will determine the physical structure of TDW and DL and subsequently evaluate the performance of the physical design. In order to guarantee the explainability design goal, we should observe the following steps during the implementation phase:

1. Finish the implementation tasks in the horizontal pipeline in the first place, then switch to finish the tasks in the vertical pipeline.
2. In the horizontal pipeline, DIKW provenance is among the top priorities to implement since DIKW provenance acts as a backbone for the explainability of ProtoDLS, and meanwhile the technologies behind it are relatively mature and engineering oriented.
3. In the vertical pipeline, mathematical formulas and algorithms are the first two implementation factors before we start implementing engineering-related factors.
4. In the last step, finish the implementation tasks for multimodality explainability.

ProtoDLS is an ambitious and challenging project with uncertain risks, which requires a continuous investment of capital and human resources. Only after a process of thoughtful and considerable design and implementation, it is estimated that ProtoDLS will reach a preliminary stage in 10 months and implement a primary overall explainability. At that stage, compared with some popular data lake systems on the market, such as Apache Hudi (2020), Apache Iceberg (2020), Apache Kudu (2020), Aws Data Lake (2020), and Delta Lake (2020) ProtoDLS will gain some competitive advantages, as illustrated in Table 1.

**TABLE 1:** Feature comparison with some popular data lake systems on the market.

	**Delta Lake**	**Apache Iceberg**	**Apache Hudi**	**Apache Kudu**	**AWS Data Lake (Dremio)**	**ProtoDLS**
Hadoop support	√	√	√	–	–	√
Metadata management	√	√	√	√	√	√
Workload management	–	–	–	–	√	√
Data governance	–	–	–	–	√	√
Streaming	√	√	√	√	√	√
Versioning	√	–	√	–	√	–
Spark SQL	√	–	√	√	–	–
Index	–	√	√	√	√	√
Row-level update	√	√	√	√	√	–
ACID transactions	√	√	√	√	√	–
Standard compliance	–	–	–	–	√	–
Security	–	–	–	√	√	√
S3 support	√	√	–	–	√	–
Explainability	–	–	–	–	–	√

## Conclusion and Future Work

The large amounts of data continuously generated from heterogeneous types of biological resources cause great challenges for advancing biological research and development; accordingly, these challenges will further incur great difficulties for biological data processing subsequently. To attack these challenges, this paper presents a design scheme for constructing a practical data lake platform for processing multimodal biological data using a prototype system named ProtoDLS. Explainability is a major concern when we deploy and use such a platform oriented for processing of biological resources, ProtoDLS adopts a dual mechanism to ensure explainability across the platform. On the horizontal landscape, ProtoDLS ensures the intra-component explainability from data acquisition to data presentation. On the other hand, on the vertical axis, ProtoDLS ensures the inner-component explainability including mathematics, algorithm, execution time, memory consumption, network latency, security, and sampling size.

The explainability is a rather broad concept, with multiple meanings in diverse scenarios, in a degree, to realize a full spectrum of explainability is somewhat close to the realization of artificial general intelligence (AGI), which will cost substantial human resources and capital investment. Also, the design of ProtoDLS is only a little step toward this. So many aspects need to be considered for ProtoDLS. For example, to design a typed DIKW resource framework will stand on a more abstract level to explain DIKW provenance, which will enhance the degrees of explainability on the horizontal axis in ProtoDLS. Every vertical module of each component leaves a huge gap for further fine-tuning that will require considerable research efforts and sometimes need several times of practical experiments. Finally, we should start from the logical prototype design given by this paper and begin implementing some subsets of ProtoDLS. For example, with the help of NLP techniques, an extensible and highly concurrent metadata management component can be designed and implemented, with a dialogue module supporting human understandable sentences. Upon the submission of this paper, the physical design of ProtoDLS has already started off, and implementation also has initiated simultaneously to prepare some initial verification.

To the best of our knowledge, this may be the first time that a logical design of a prototypical data lake is proposed in terms of the explainability around the data processing in a data lake. Although this paper is relatively elementary, we also hope to provide a starting point and a stepping stone for any academic researchers and industrial practitioners in bioinformatics, genetics, and phenomics, or people interested in data lake research and deployment in any other fields. For people who are doing research on the data lake explainability, this paper also may be beneficial and helpful.

## Data Availability Statement

The original contributions presented in the study are included in the article/supplementary material, further inquiries can be directed to the corresponding author.

## Author Contributions

HC proposed the conceptual framework, designed the initial version of the prototype system, revised the manuscript, and provided the final submission version. YD corrected the conceptual framework, provided some insightful contributions to the design details of ProtoDLS, and proposed an upgraded version of it. HC and YD edited and modified the manuscript, figures, and table. Both authors contributed to the article and approved the submitted version.

## Conflict of Interest

HC was employed by Great Wall Motors company at the time of the study. The company was not involved in the study design, collection, analysis, interpretation of data, the writing of this article or the decision to submit it for publication. The remaining author declares that the research was conducted in the absence of any commercial or financial relationships that could be construed as a potential conflict of interest.

## Abbreviations

AGI: Artificial general intelligence
AI: Artificial intelligence
AM: Algorithm metadata
AV: Algorithm visualization
BioBPX: Biological Pathway Exchange
DG: Data governance
DI: Data ingestion
DIKW: Data–information–knowledge–wisdom
DL: Data lake
DM: DIKW metadata
DP: Data pond
DPV: DIKW provenance visualization
DS: Dialogue system
EI: Explainable infrastructure
ELM: Extreme learning machine
EML: Explainable machine learning
HCLS: Health care and life sciences
IM: Infrastructure metadata
IV: Infrastructure visualization
JS: Job scheduler
KG: Knowledge graph
LIME: Local Interpretable Model-agnostic Explanations
MC: Metadata catalog
ML: Machine learning
MM: Mathematics metadata
NA: Narrator
PCA: Principal component analysis
ProtoDLS: Prototypical Data Lake System
SMV: Software Metrics Visualization
SP: Security and privacy
SRM: Software runtime metrics
ST: Sandbox training
SVD: Singular value decomposition
TA: Twin agent
TDW: Traditional data warehouse
VI: Visualization
XAI: Explainable artificial intelligence.
